# Reconstruction of the Drive Underlying Food Intake and Its Control by Leptin and Dieting

**DOI:** 10.1371/journal.pone.0074997

**Published:** 2013-09-25

**Authors:** Johan Grasman

**Affiliations:** Biometris, Wageningen University and Research Centre, Wageningen, The Netherlands; Hosptial Infantil Universitario Niño Jesús, CIBEROBN, Spain

## Abstract

The intake of food and the expenditure of calories is modelled by a system of differential equations. The state variables are the amount of calories stored in adipose tissue and the level of plasma leptin. The model has as input a drive that controls the intake of food. This drive consists of a collective of physiological and psychological incentives to eat or to stop eating. An individual based approach is presented by which the parameters of the system can be set using data of a subject. The method of analysis is fully worked out using weight data of two persons. The model is prone to extensions by transferring incentives being part of the input to the collection of state variables.

## Introduction

In psychology the notion of *drive* was introduced by Woodworth [1]. It is defined as an urgent need that strives for expression. This need can be rooted in some type of physiological deficiency, such as hunger, or be purely of mental origin, e.g. in the form of some compulsive thought. A drive forces a person to act; this study deals with the action of taking in food. The decision to start or stop eating can simply be governed by a daily routine, but in practice, especially for people living in a western culture, the decision on what and how much to eat may get rather complex [2], [3]. This study deals with a mathematical model that describes the balance between taking up calories and their expenditure for the purpose of maintaining the body and undertaking actions. The model consists of a system of differential equations for a set of state variables being physiological quantities. The minimum of two state variables is taken: the amount of calories *C* stored in body fat tissue and the level *L* of the hormone leptin in the blood circulation. The change in *C* follows directly from the difference between the intake and the expenditure of calories. The goal is to fit the model to available data on food intake. For that purpose two long-term time series on body weight are taken in consideration. This form of data-directed modelling limits the use of highly complex model systems.

As remarked the intake of calories requires attention in order to stay healthy. This was also the case in prehistoric times, but for a different reason than in present days: surviving was the main issue. Just eating for killing the hunger of the moment was not wise. It was better to eat some more to overcome possible hardships in later days. Maybe we still carry this trait in our genes which makes us consciously or unconsciously decide to store a sufficient amount of energy in our body [4]. In fact the genetics of the physiological side of energy dynamics of modern western individuals still does not differ significantly from people that continued to live as hunter-gatherers [5]. The intention to eat more than necessary for the moment, as suggested above, may be a persistent relic from the past. The drive of eating more than is momentarily needed requires some control. This is, amongst others, provided by the hormone leptin: the hypothalamus registers how many calories one still has in storage by reading off the concentration of leptin in the blood [6]. In the model this hormone, produced in adipose tissue, is taken as the only steering mechanism. Nevertheless other factors are still accounted for in the model through the way they may influence the drive for food; e.g. insulin which, similar to leptin, may slow down the intake of food [7]. From the gastrointestinal tract a signal (the hormone cholecystokinin) is sent out in case of satiety and after a fasting time the drive to eat is stimulated by the hormone ghrelin produced in the stomach and the pancreas [8]. Next to these physiological processes also mental states may stimulate or slow down the intake of calories; examples are the consumption of comfort foods [9] and the choice of whether or not to follow a diet for some period [10]. With the drive *D* defined in this way this study concretizes the hypothesis underlying a general model of intake regulation as formulated by Speakman et al. [11]: “food intake results from the net sum of the activity of all of the compensated and uncompensated factors acting simultaneously”. The drive *D* is reconstructed from the model by posing a number of assumptions. Most importantly it is assumed that weight increase (or decrease) is due to decreased (increased) calorie expenditure. Furthermore, rate coefficients in the two differential equations are estimated by using quantitative results from the literature. This differential equation approach of describing food intake and the corresponding energy flow was also followed by Chow and Hall [12]. Their type of modelling is purely physiologically oriented and more detailed, while here a connection with mental processes is made. In [13] a similar approach has been followed to model the intake of nicotine.

**Figure 1:**
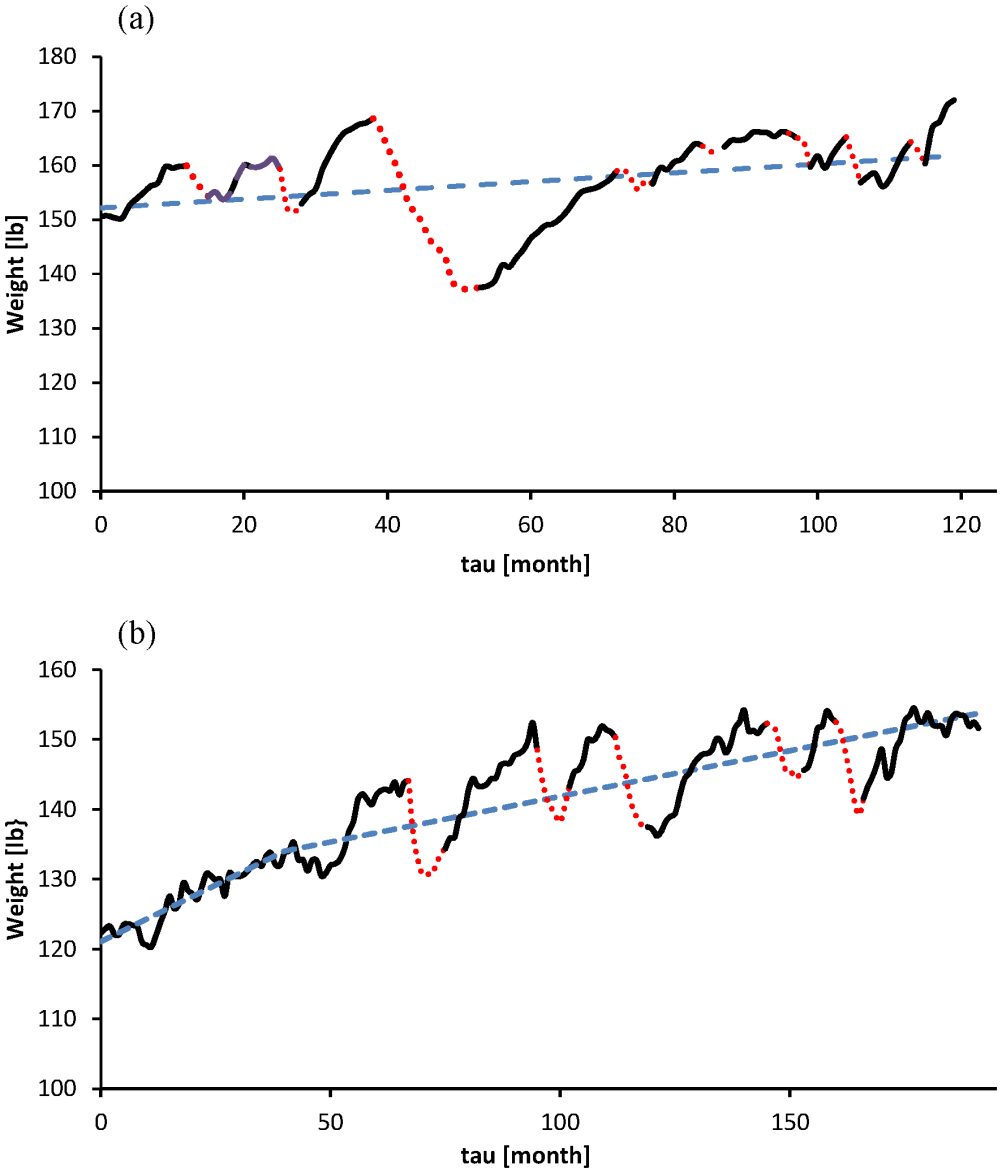
History of weight fluctuations of two adult females from data presented in [15]. During the period the subjects were taking part in dietary programs (dotted intervals). The dashed lines represent the weight for constant parameter values, see Table 1: (a) Subject A with a mean weight *W*_mean_ = 157.0 [lb]. (b) Subject B with *W*_mean_ = 140.8 [lb]. This history has been split in two sections: up to month 40 with *W*_mean_ = 127.4 [lb] and a second section with *W*_mean_ = 144.4 [lb]. From the first section all parameters are estimated. In the second section these estimates are used again except for the rate of the constant relative decrease ε of calorie expenditure which was estimated anew.

It is understood that the pattern of food intake can be quite diverse [14]. A large part of the literature on this topic is devoted to extreme situations due to physiological or psychological deficiencies. This study only aims at individuals not suffering from serious eating disorders. Still the remaining group keeps showing a variety of feeding traits. Looking to weight histories of individuals we see different patterns of weight change through the years. The case of yoyo dieting [3] is a striking example of such a pattern. Due to the large diversity of fluctuations it has no use to construct a model representing a prototype of eating pattern showing the ideal dynamics; we have to perform individual based analyses. This study takes the weight history of two subjects [15] as starting point, see Figure 1.

## Materials and Methods

The dynamical system under consideration consists of two state variables: the calories *C* [cal] stored in adipose tissue and the concentration *L* [ng ml^−1^] of plasma leptin. The assumption that the dynamics is linear leads for the change in time *t* [day] of the state variables to the following differential equations 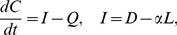
(1ab)

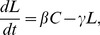
(1c) where *I* [cal day^−1^] denotes the intake rate of calories and *Q* [cal day^−1^] the expenditure rate due to body maintenance and physical action. The intake rate *I* follows from the drive *D* damped by the effect of leptin.

If one rejects the inclusion of the drive component, then formula (1b) is void, so that Eq.(1a) becomes merely a matter of book keeping of calories while Eq.(1c) only gives an account of the presence of leptin in the blood circulation. However, the aim of this study is to analyse the effect of leptin upon the intake of food [8], [16]–[17]. The introduction of the drive *D*, being composed out of a number of incentives to eat and not to eat, facilitates in a simple manner the inclusion of the feedback role of the hormone leptin in the process of taking up calories.

**Figure 2:**
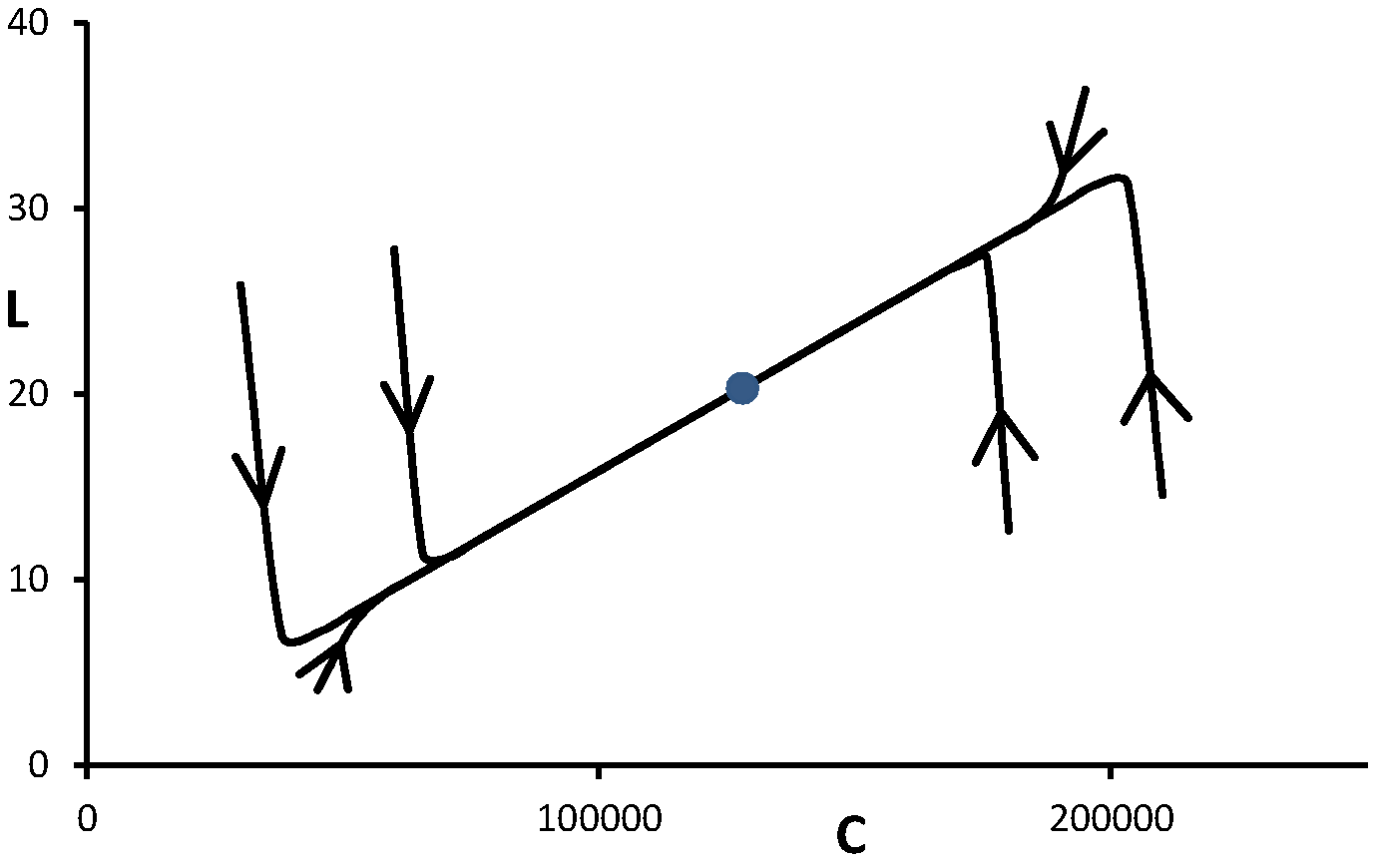
Dynamics of the system of differential equations (1abc) for the (constant) parameter values given in Table 1. It is noted that the two differential equations have different time scales. Eq.(1c) has a fast time scale meaning that in the beginning *L* rapidly changes its value so that the system reaches a quasi-stationary state satisfying 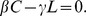 Next along this line the equilibrium (*C*, *L*) is slowly approached in a way prescribed by Eq.(1a) with constraint (2).

The first task in dealing with the model (1abc) is to find values for the parameters. Since the level of leptin accommodates more quickly than the weight, see Figure 2, it may be assumed that the system is in a quasi-steady state with
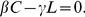
(2)


The mean concentration of plasma leptin in women is estimated *L* = 20.3 [ng ml^−1^], see [18]. In this publication it is also found that the average percentage of body fat δ in humans is around 30%. Thus, if 1 [lb] of body fat is equivalent to ρ = 3500 [cal], then the approximation 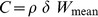
(3) can be made. In [6] it is reported that Leptin is taken out of the blood circulation by the kidneys with a halftime of 24.9 [min]. It is remarked that the data refers to males only. From results of an experimental study [19], in which additional leptin is administered to both males and females, it can be concluded that for females the value does not differ significantly. Thus, the parameter γ takes the value 40.1 [day^−1^]. Together with Eqs.(2)–(3) it yields 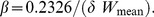
(4)


The expenditure of calories may vary in time. It is in the order of 2000 calories per day (www.livestrong.com). It is also supposed that the gain in weight through the years is not caused by an increase of the intake of calories but merely by a decrease of physical exercise [20]. The relative expenditure of calories is assumed to decrease at a constant rate ε, while the drive *D* is also set at a constant value. The parameter ρ is kept at the value given above in agreement with the literature; the value is independent of the subject under consideration. In the quasi-steady state with condition (2) the differential equation (1a) becomes
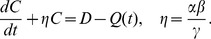
(5ab)


The assumption that the relative decrease of calorie expenditure is constant gives that
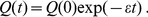
(6)


The solution of (5a) reads
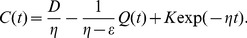
(7)


The integration constant *K* can be set zero provided that η>>ε; in that case the influence of the starting value is not felt anymore (quasi-steady state). In order to use the weight data we replace the variable stored calories *C* by the weight *W*:
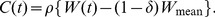
(8)


The parameters *D*, *Q*(0), δ, η and ε are estimated by minimizing the residual sum of squares of the difference between observation *W_i_* and model value *W*(*t_i_*). From their values the parameters α and β can be computed.

In a next step Eq.(5a) is considered again, but now *D* is let free to change in time. Furthermore, the time scale of months is chosen and the transformation (8) is used. In this way the evolution of the drive *D* is reconstructed by using the weight data and the estimated parameters, see Appendix S1. Discretization of Eq.(5a) yields with the use of (6):
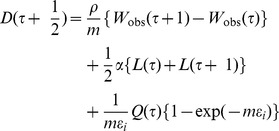
(9) with the time τ in months and with *L*(τ) depending on *W*_obs_(τ) as given by (2) and (8).

## Results

Application of nonlinear regression based on least squares (Matlab: lsqnonlin) leads to the estimates given in Table 1, see Program S1 for the details. For subject B the trend in weight increase shows an abrupt change after 40 months. For this subject the parameters are estimated for the time interval preceding this transition point. For the time interval beyond this point the parameters have been giving the same value except for ε_1_. While estimating the parameter (ε_2_) for that interval one has to impose a continuity condition upon *Q* at the point of transition. In Figure 1 the regression curves are depicted.

**Table 1:** Parameter estimates based on the weight data.

	*Q*(0)	β	δ	η	α	*D*	ε_1_	ε_2_
A	2571	3.68×10^−3^	0.405	2.78×10^−5^	0.303	2586	1.00×10^−7^	
B	2100	5.78×10^−3^	0.316	1.17×10^−4^	0.816	2152	2.06×10^−6^	8.30×10^−7^

It is found that the reconstructed drive *D*(τ) fluctuates around a constant level (in the reconstruction it is allowed to change freely; only for the sake of estimating the parameters constancy was assumed). For subject A (B) the regular *D*-values, outside the dietary periods, have a mean 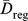 of 2210 (2110) [cal day^−1^] with a standard deviation of 131 (183). Also for each of the dietary periods the mean difference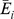 with 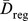 was computed, see Table 2. In a one sample T-test with these *E*-values a significant positive effect of dieting was found with *p* = 0.000 (0.009). In Table 3 the effect of dieting is compared with that of leptin (α*L*).

**Table 2:** Mean value 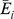 [cal day^−1^] of the effect of dieting upon the drive *D*.

subject	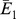	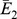	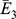	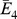	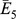	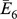	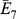	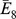	Mean	St.dev.
A	263	346	323	120	181	342	369	193	267	87
B	77	66	228	151	274				160	82

**Table 3:** Effect of leptin and dieting with respect to the mean drive outside the dietary periods.

subject	dieting	leptin
A	12%	0.3%
B	7%	1%

**Figure 3:**
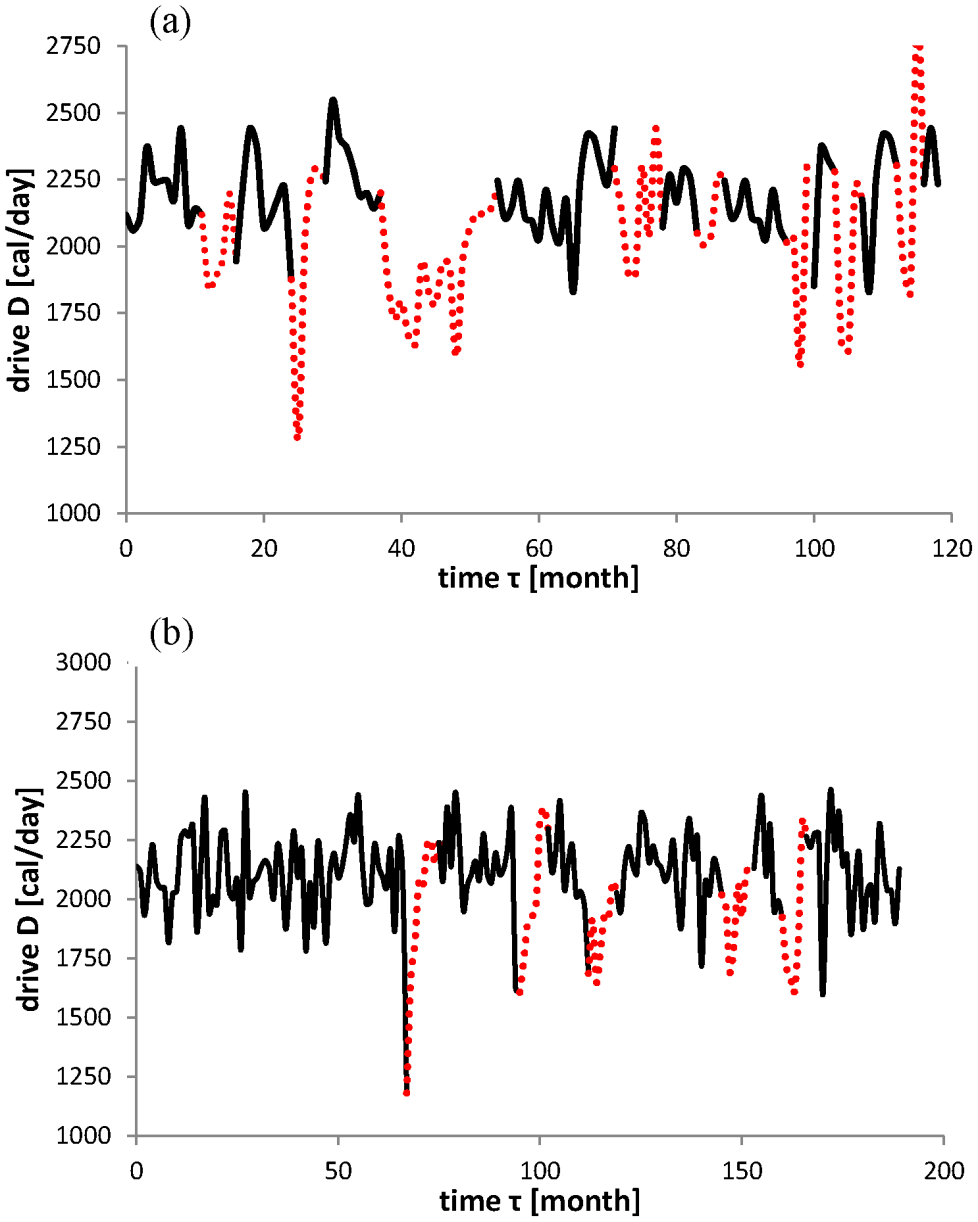
Reconstruction of the drive *D* (solid line) containing dietary periods (dotted). (a) Subject A. (b) Subject B.

**Figure 4:**
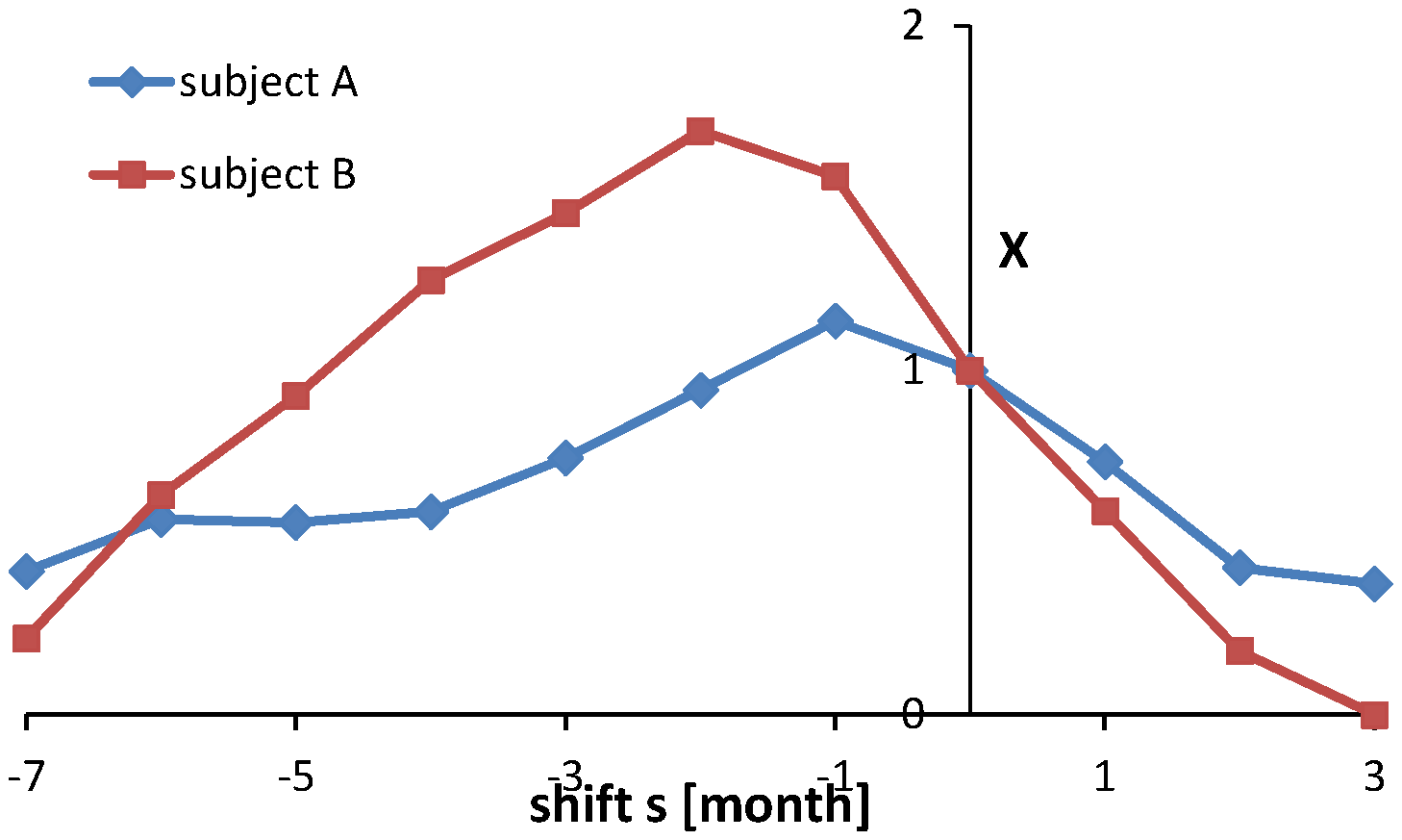
Cross-correlation *X*(*s*) of the function *E*(τ) given by (10) and the block function *B*(τ) which has the value 1 during dietary periods and 0 outside these periods.

Thus, during dieting the drive *D* is brought down. In Figure 3 it is seen that this tendency is already present before the start of the dietary period, while it goes up again before the end of the period. Apparently, the (lack of) motivation to lose weight causes this shift backward in time of the effect of the dietary process upon the drive. It is quantified by introducing of the difference between 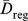 and the actual drive over the full time period
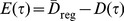
(10) and its cross-correlation *X*(*s*) with the block function *B*(τ) with *B*(τ) = 1 during the dietary periods and 0 outside these periods. The variable *s* denotes a time shift with *B*(τ) shifted in time over an interval *s* (backwards for *s*<0). The function *X*(*s*) is normalized such that *X*(0) = 1, see Figure 4.

## Discussion

Many studies have appeared on obesity and deal with the question why do people eat too much, or in the case of anorexia why are other people fasting in such an extreme way [21]. This study aims at the problem that precedes these questions: which are the incentives to start or stop eating for any person? Such a question is not answered by giving detailed accounts on the intake of food and the expenditure of energy: people will not accurately calculate how much they should eat given their planned physical efforts. Fortunately we have physiological controls to our disposal that may stimulate us to eat less or more in order to stay in the best condition. In addition we may consciously decide to follow some diet for our well-being. These controls steer our intake of food and are part of the models explaining decisions on food intake.

The notion of *drive* was introduced as a collective noun for all incentives that apply to the intake of food that are not explicitly contained in the model. One of them was following a diet, while the effect of leptin also being a stimulus not to eat was contained in the model. As a result of this investigation one may quantify and thus also compare the way both leptin and dieting diminish the intended intake of food, see Table 3. In a subsequent study dieting can be taken as part of the model by replacing in (1a) the food intake by *I* = *D*−*E*−α*I*, where *E* denotes the amount by which the intended intake rate is reduced from dieting. This process of subsequently extending the model can be continued by including a next incentive in the model e.g. the hormone ghrelin which stimulates eating. In this way step by step the drive *D* can be peeled off.

The dynamics of the differential equation model (1abc) has two time scales: one in the order of minutes in which the system may reach the quasi-steady state. In this state the leptin concentration follows the calorie content of the adipose tissue. In a time scale of the order of days the system adapts to changes in the food intake. For the problem of structural weight change there is still a scale of months to years in which some trend is expressed. The system, as we have formulated, does still produce a meaningful result at this scale because of the condition that has been imposed upon the long term expenditure of calories. Ravussin and Bogardus [20] confirmed that weight increase in the process of aging can be caused by a decrease of calorie expenditure. They reported that in addition the food intake may go down although at the same time the weight is increasing. A trend that is also found in the present results. An increase in weight over a short period is no indication for an increase in the long term: it has already been observed by Pearcey and De Castro [22] that the intake of calories over a week of persons with differing weights does not differ much. On the other hand weight gain over a week was shown to be strongly correlated to calorie intake in that week. Clearly, in order to get grip on durable weight change our model needs more long term information on calorie intake and expenditure of the person under consideration. This necessity of studying specifically the long term dynamics of the energy balance was also noted in [12].

Weight fluctuations from dieting represent a type of relaxation oscillation [23] exhibiting a sudden change when some threshold is exceeded. In this case the threshold is formed by an unacceptably high weight. This is a form of set point regulation which in the literature previously was attributed to the way physiologically the amount of body fat is controlled [11]. In the reconstructed drive it is observed that effectively the dieting process exhibits a shift in time: one or more months before the formal dietary period starts subjects are already bringing down their eating drive and well before the end of the period it is going up again, see Figure 4 for a quantitative analysis of this phenomenon.

The linear model (1abc) does not suffice to analyse the dynamics of subjects with an eating disorder and/or deficiencies in their metabolism. In order to account for the effect of such a malfunctioning, nonlinear elements should be included. An example is the lower sensitivity of the brain for high leptin concentrations caused by an extended period of overweight [24]. It is remarked that for such a nonlinear model with additional parameters the estimation of parameters for the present highly fluctuating data fails. This is due to the fact one cannot use anymore an explicit solution for the quasi-steady state as given by (7) with *K* = 0. Then parameter estimate methods for differential equations have to be used. The Matlab package Grind (www.sparcs-center.org/grind) provides the procedure “optimpars” being a suitable tool for such an investigation in a more extended version of the present model. Another limitation of the present study is the number of only two subjects. From the results presented in Program S1 and in the Tables it cannot be concluded how the values of the different parameters are distributed in a (sub)population of individuals.

## Supporting Information

Appendix S1 **Reconstructing the drive** ***D*** **.**
(DOCX)

Program S1 **Parameter estimates.**
(XLSX)
