# Supplementary material for: Reconstruction of the Drive Underlying Food Intake and Its Control by Leptin and Dieting
Source: PLoS One. 2013 Sep 25;8(9):e74997. doi: 10.1371/journal.pone.0074997 (PMC3783460; doi:10.1371/journal.pone.0074997)
Supplement: Appendix S1 — Reconstructing the drive D . (DOCX) [file pone.0074997.s001.docx]

**Appendix S1**

**Reconstructing the drive**

The drive *D*(*t*) follows from the differential equation

$\frac{dC}{dt}=D(t)-\alpha L(t)-Q(t),$ (A1)

with constraint

$\beta C-\gamma L=0$. (A2)

Eq.(A1) is transformed into a differential equation for *W*(*t*) using

$C(t)=\rho\{W(t)-(1-\delta)W_{\text{mean}}\}$ $(\text{A}3)$

and integrated over the time interval [*m*τ, (*m* + 1)τ], τ = 0, 1, …, *T*_1_:

$$\int_{m\tau}^{m(\tau+1)} \rho\frac{dW(t)}{dt}dt=\int_{m\tau}^{m(\tau+1)} D(t)dt -\int_{m\tau}^{m(\tau+1)} \alpha L(t)dt -\int_{m\tau}^{m(\tau+1)} Q(t)dt.$$

In this equation *Q*(*t*) = *Q*(*m*τ) exp{-ε(*t - m*τ)}. The left hand side and the last term at the right hand side can be integrated exactly. The first term in the right hand side is approximated by substituting the middle value for the integrand, while for the second term the trapezium rule is used. Replacing *W*(*t*) by the observed values and using the time scale of months we obtain:

$\begin{matrix} D(\tau+ \frac{1}{2})=\frac{\rho}{m}\{W_{\text{obs}}(\tau+1)-W_{\text{obs}}(\tau)\}+\frac{1}{2}\alpha\{L(\tau)+L(\tau+ 1)\}+\frac{1}{m\varepsilon}Q(\tau)\{1-\exp(-m\varepsilon)\}. \tau=0,...,T_{1}-1. \\ \end{matrix}$Note that in this equation *L*(τ) is replaced by *W*_obs_(τ) using (A2)-(A3). The parameter values are given in Table I. For subject B the parameter ε takes different values for the time intervals before and after the point τ = 40.
